# Infections with Spore-forming Bacteria in Persons Who Inject Drugs, 2000–2009

**DOI:** 10.3201/eid1901.120044

**Published:** 2013-01

**Authors:** Norah E. Palmateer, Vivian D. Hope, Kirsty Roy, Andrea Marongiu, Joanne M. White, Kathie A. Grant, Colin N. Ramsay, David J. Goldberg, Fortune Ncube

**Affiliations:** Author affiliations: Health Protection Scotland, Glasgow, Scotland, UK (N.E. Palmateer, K. Roy, C.N. Ramsay, D.J. Goldberg);; Health Protection Agency, London, UK (V.D. Hope, A. Marongiu, J.M. White, K.A. Grant, F. Ncube);; London School of Hygiene and Tropical Medicine, London (V.D. Hope)

**Keywords:** Substance abuse, intravenous, clostridium, botulism, tetanus, anthrax, spores, bacteria, persons who inject drugs, PWID

## Abstract

Since 2000 in the United Kingdom, infections caused by spore-forming bacteria have been associated with increasing illness and death among persons who inject drugs (PWID). To assess temporal and geographic trends in these illnesses (botulism, tetanus, *Clostridium novyi* infection*,* and anthrax), we compared rates across England and Scotland for 2000–2009. Overall, 295 infections were reported: 1.45 per 1,000 PWID in England and 4.01 per 1,000 PWID in Scotland. The higher rate in Scotland was mainly attributable to *C. novyi* infection and anthrax; rates of botulism and tetanus were comparable in both countries. The temporal and geographic clustering of cases of *C. novyi* and anthrax into outbreaks suggests possible contamination of specific heroin batches; in contrast, the more sporadic nature of tetanus and botulism cases suggests that these spores might more commonly exist in the drug supply or local environment although at varying levels. PWID should be advised about treatment programs, injecting hygiene, risks, and vaccinations.

## Abstract 2

Clusters of almost 300 cases in time and location might be the result of contamination of specific heroin batches.

*Clostridium* and *Bacillus* spp. produce spores that can be found in soil, dust, human and animal intestines, and aquatic environments; these spores can remain viable for long periods (*1*). Spores can contaminate illicit drugs or drug-injecting equipment. If injected intravenously, intramuscularly, or subcutaneously, spores can germinate and produce potent neurotoxins or histotoxins that cause illness and death (*2*). In persons who inject drugs (PWID), these organisms often initially cause localized infections; however, the toxins they produce can result in severe systemic illness, which usually becomes apparent within a week after infection.

Infections with spore-forming bacteria in PWID have historically been more common in the United States than in Europe. By the 1950s, injection drug use accounted for most cases of tetanus in New York (*3*,*4*), and wound botulism associated with injecting black tar heroin was first described in California just over 2 decades ago (*5*). In contrast, such infections have occurred more recently in Europe; in the United Kingdom, for example, few infections had been reported before 2000 (*1*). Nevertheless, a recent article noted that 367 infections with spore-forming bacteria among PWID in Europe were reported during 2000–2009 (*6*). Although high rates of these infections were reported in northwestern Europe (United Kingdom, Norway, and Ireland), few cases have been reported elsewhere in Europe. The reasons for this marked regional variation within Europe remain unclear but might reflect drug trafficking routes, the type of drugs injected locally, and/or differences in local injecting practices (*6*).

In addition to the varied extent of these infections among PWID across Europe, some regional variation within the United Kingdom has been noted (*7*) but not fully explored. To further explore this variation, we compared the regional rates of infection and death caused by a small number of aerobic and anaerobic spore-forming bacteria among PWID in Scotland and England over a 10-year period beginning in 2000. The availability of detailed epidemiologic data on cases in England and Scotland enabled us to examine regional and temporal trends and demographic patterns. Information about differences in drug-injecting populations and practices that might be associated with infection could be used to prevent future infections.

## Materials and Methods

### Case Ascertainment

We collated information about reported cases of infection with *Clostridium botulinum* (botulism), *C. tetani* (tetanus), *C. novyi*, and *Bacillus anthracis* (anthrax) among PWID in England and Scotland with dates of onset from January 2000 through December 2009. Information about suspected cases of botulism or tetanus was obtained from voluntary or statutory notifications to the Health Protection Agency and Health Protection Scotland; reports included information about possible risk factors. Corresponding samples were sent to the Foodborne Pathogens Reference Unit, the Special Pathogens Reference Unit, or the Anaerobic Reference Laboratory for the detection of toxin and microbiological confirmation. Confirmation criteria have been described (*8*,*9*). Clinical, demographic, and risk factor information was obtained from a questionnaire administered to patients by clinicians or microbiologists. Information about cases of *C. novyi* infection and anthrax were obtained from reports and documentation of the respective outbreaks (*7*,*10*–*12*); case definitions are described in these reports. The analyses presented here are limited to definite and probable *C. novyi* infections and confirmed anthrax cases.

### Data Analysis

To derive infection rates, we used regional estimates of the number of PWID in England (2004–05 fiscal year) and Scotland (2006), closest to the midpoint of the 10-year period (2000–2009) (*13*,*14*). Both sets of estimates of PWID populations were derived by log–linear modeling of capture–recapture data. Numbers of infections were tabulated by region (England) and National Health Service Board area (Scotland), and rates per 1,000 PWID were calculated.

Numbers of infections were also tabulated by sex, and median age of case-patients was calculated. To compare demographics, we compared the sex distribution and median age of our study population with that derived from national surveys of PWID in England and Scotland (these data were not available from the capture–recapture PWID estimates described above) undertaken in years closest to the midpoint of the 10-year period. For England, we used data from the 2005 Unlinked Anonymous Monitoring Survey of PWID (*15*) and, for Scotland, the 2008–2009 Needle Exchange Surveillance Initiative (*16*). These 2 surveys aimed to recruit representative samples of PWID in contact with specialist services; the numbers of PWID participating in these surveys who had injected in the preceding 4 weeks were 1,740 and 1,772, respectively. We compared national survey respondents and case-patients in terms of sex and age by using χ^2^ tests (or Fisher exact tests when there were <5 persons in a given tabular cell) and Wilcoxon rank tests, respectively.

## Results

During January 1, 2000–December 31, 2009, a total of 295 infections caused by spore-forming bacteria (157 botulism, 33 tetanus, 92 *C. novyi*, and 13 anthrax) were reported among PWID in England and Scotland; the overall infection rate was 1.83 cases per 1,000 PWID. Two thirds (199) of these cases were reported in England and one third (96) in Scotland, corresponding to rates of 1.45 and 4.01 per 1,000 PWID, respectively (Table 1). 

**Table 1:** Cases of infection with spore-forming bacteria and rates of infection among PWID, by health region, England and Scotland, 2000–2009*

Health region	No. PWID†	No. cases (rate/1,000 PWID)				Total no. cases (rate/1,000 PWID, 95% CI)
		Botulism	Tetanus	*Clostridium novyi* infection	Anthrax	
England	137,141	139 (1.01)	28 (0.20)	32 (0.23)	0	199 (1.45, 1.22–1.65)
East of England	9,418	17 (1.81)	0	2 (0.21)	0	19 (2.02, 1.45–3.04)
East Midlands	11,796	15 (1.27)	4 (0.34)	1 (0.08)	0	20 (1.70, 1.48–1.91)
London	17,909	28 (1.56)	4 (0.22)	2 (0.11)	0	34 (1.90, 1.42–2.10)
North East	8,959	6 (0.67)	1 (0.11)	0	0	7 (0.78, 0.66–0.99)
North West	22,089	14 (0.63)	7 (0.32)	16 (0.72)	0	37 (1.68, 1.47–1.97)
South East	13,778	9 (0.65)	3 (0.22)	5 (0.36)	0	17 (1.23, 0.95–1.41)
South West	17,444	23 (1.32)	3 (0.17)	1 (0.06)	0	27 (1.55, 1.38–1.69)
West Midlands	14,734	4 (0.27)	5 (0.34)	1 (0.07)	0	10 (0.68, 0.59–0.74)
Yorkshire and the Humber	21,014	23 (1.09)	1 (0.05)	4 (0.19)	0	28 (1.33, 1.23–1.41)
Scotland‡	23,933	18 (0.75)	5 (0.21)	60 (2.51)§	13 (0.54)	96 (4.01, 3.43–4.29)
Ayrshire and Arran	2,373	0	0	0	0	0
Borders	201	0	0	0	0	0
Dumfries and Galloway	486	0	1 (2.06)	0	0	1 (2.06, 1.49–2.70)
Fife	1,270	2 (1.57)	0	2 (1.57)	1 (0.79)	5 (3.94, 3.27–4.64)
Forth Valley	786	1 (1.27)	0	0	0	1 (1.27, 1.04–1.52)
Grampian	3,056	6 (1.96)	2 (0.65)	3 (0.98)	0	11 (3.60, 2.83–4.48)
Greater Glasgow and Clyde	8,862	8 (0.90)	1 (0.11)	50 (5.64)	9 (1.02)	68 (7.67, 5.74–9.17)
Highland	734	0	0	0	0	0
Lanarkshire	1,649	0	0	2 (1.21)	2 (1.21)	4 (2.43, 1.92–4.46)
Lothian	3,262	0	1 (0.31)	0	0	1 (0.31, 0.23–0.40)
Tayside	1,254	1 (0.80)	0	0	1 (0.80)	2 (1.59, 1.26–1.97)

*PWID, persons who inject drugs. †References *13,14*. ‡Regions of mainland Scotland only. §Region not known for 3 of the 60 case-patients who had *C. novyi* infection.

**Figure 1:**
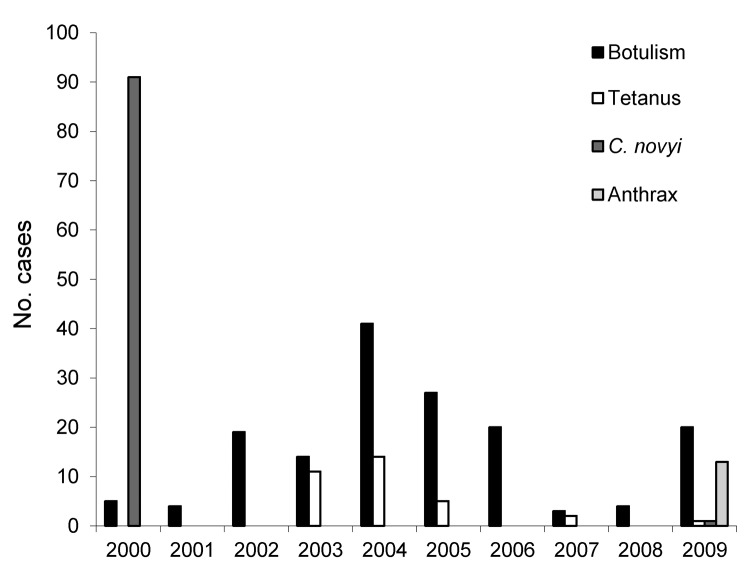
Annual numbers of cases of botulism, tetanus, *Clostridium novyi* infection, and anthrax among persons who inject drugs, England and Scotland, 2000–2009.

The number of reported cases varied over time (Figure 1). The *C. noyvi* infections and anthrax cases were clustered in 2000 and 2009, respectively, and most tetanus cases occurred during 2003–2005. By contrast, botulism was reported in all years; the annual number of cases varied from 3 to 41.

**Figure 2:**
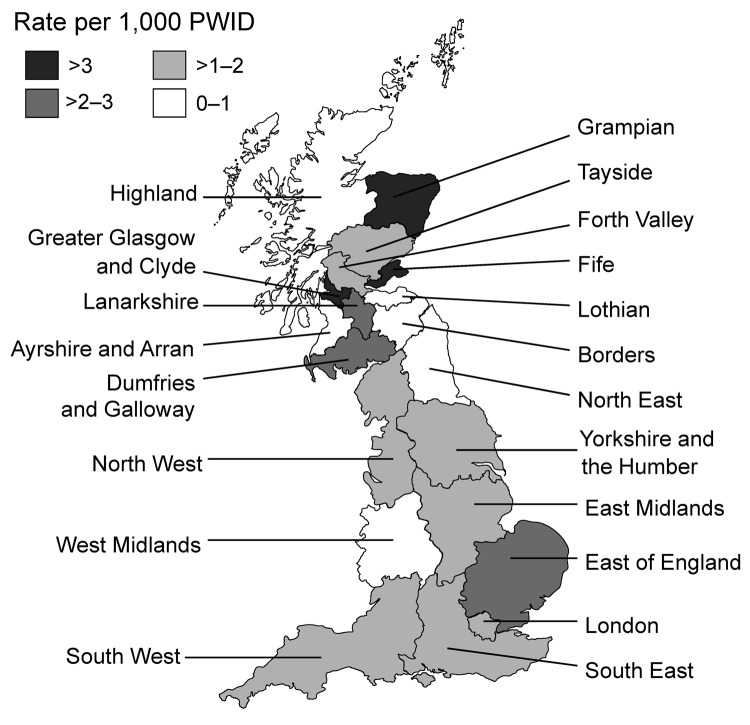
Rates of infection with spore-forming bacteria (*Clostridium botulinum*, *C.tetani*, *C. novyi*, and *Bacillus anthracis*) among persons who inject drugs (PWID), by health region, England and Scotland, 2000–2009.

Infection rates varied by health region. In England, rate of infection varied from 0.68 cases per 1,000 PWID for the West Midlands to 2.02 for the East of England (Figure 2); rates were also high for the East Midlands, London, and the North West (1.7, 1.9, and 1.7 cases/1,000 PWID, respectively). In Scotland, rates ranged from zero in 3 rural areas with small populations of PWID (Ayrshire and Arran, Borders, and Highlands) to 7.7 per 1,000 PWID in Greater Glasgow and Clyde; rates were also high in Grampian (3.6 cases/1,000 PWID) and Fife (3.9 cases per /1,000 PWID).

In terms of specific infections, the rate of botulism was slightly higher for England than for Scotland, although this difference was not statistically significant (1.0 vs. 0.8 cases/1,000 PWID, p = 0.232), and rates of tetanus were similar for both countries (0.20 vs. 0.21/1,000 PWID, p = 0.962). In contrast, rates of *C. novyi* infections and anthrax were markedly higher for Scotland than for England (2.5 vs. 0.2 cases/1,000 PWID, p<0.001; and 0.5 vs. 0 cases/1,000 PWID, p<0.001, respectively). *C. novyi* infections were particularly concentrated in Greater Glasgow and Clyde (5.6 cases/1,000 PWID) and in the North West region of England (0.7 cases/1,000 PWID). Higher than average rates of botulism were reported in the East of England region (1.8 cases/1,000 PWID) and in Grampian (2.0 cases/1,000 PWID).

When we compared the demographic characteristics of case-patients with those of PWID participating in the 2 national surveys, we found that the proportion of female patients with tetanus, *C. novyi* infection*,* and anthrax was higher (38%–60%) than the proportion of female PWID in the community (24%–26%) (Table 2). These differences were statistically significant for *C. novyi* infections in England and Scotland (p = 0.011 and p<0.001, respectively) and for tetanus cases in England. In England, the median age of PWID with botulism, tetanus, and *C. novyi* infection ranged from 33 to 37 years; this age range was higher among those with botulism than among the PWID participating in the Unlinked Anonymous Monitoring Survey (37 vs. 32 years; p<0.001). In Scotland, the median ages of PWID with botulism, *C. novyi* infection, and anthrax were comparable to the median age of PWID from the community sample; whereas, the median age was higher for PWID infected with tetanus (47 vs. 33 years), although not significantly so (p = 0.065).

**Table 2:** Sex distribution and median age of PWID with botulism, tetanus, *Clostridium novyi* infection, and anthrax among a community sample of PWID, England and Scotland, 2000–2009*

PWID	England							Scotland					
	No.†	Female, %	p value‡	No.†	Median age, y (IQR)	p value§		No.†	Female, %	p value‡	No.	Median age, y (IQR)	p value§
Community¶	1,732	24		1,711	32 (11)			1,763	26		1,772	33 (10)	
With botulism	139	30	0.157	128	37 (13)	<0.001		18	11	0.186	13#	31 (5)	0.670
With tetanus	28	46	0.006	28	35 (19)	0.067		5	60	0.112	5	47 (17)	0.065
With* C. novyi* infection	32	44	0.011	32	33 (11)	0.654		60	52	<0.001	57#	30 (8)	0.086
With anthrax		NA			NA			13	38	0.297	13	35 (9)	0.093
Total with infection	199	32	0.002	188	35.5 (13.5)	<0.001		96	43	<0.001	88	31 (9)	0.232

*PWID, persons who inject drugs; IQR, Interquartile range; NA, not applicable. †For the community samples, information about sex of 8 (England) and 9 (Scotland) persons was missing, and information about age for 29 persons (England) was missing. ‡Based on the χ^2^ test or Fisher exact test (where tabular cell counts were <5 for the difference in proportion of female case-patients with the respective infections and the community sample of PWID). §Based on the Mann-Whitney test for the difference in median age between the case-patients with the respective infection(s) and the community sample of PWID. ¶Data from the Unlinked Anonymous Monitoring Survey 2005 (*15*) for England (n = 1,740) and from the Needle Exchange Surveillance Initiative 2008/2009 (*16*) for Scotland (n = 1,772). #Complete data on age were available for 13/18 botulism case-patients and 57/60 C. *novyi* case-patients.

Of the 295 reported case-patients, 52 (18%) are known to have died. Of these, 8 (5%) died of botulism, 2 (6%) died of tetanus, 36 (39%) died of *C. novyi* infection, and 6 (46%) died of anthrax.

## Discussion

Over the decade beginning in 2000, almost 300 severe infections caused by spore-forming bacteria were reported among PWID in England and Scotland; 52 of these patients died. The distribution of the cases varied markedly between these countries. In Scotland, the number of cases was excessive relative to the estimated population of PWID when compared with England; this excess, however, is mainly attributable to an excess of *C. novyi* infections and anthrax cases. In contrast, rates of botulism and tetanus for Scotland were lower than and comparable with, respectively, those for England.

In the United Kingdom, microbiological testing has usually been unable to confirm the presence of these bacterial species in seized or surrendered heroin (*2*), although, in 2009, *C. botulinum* was isolated from 1 sample of heroin seized in Scotland (K.A. Grant, pers. comm.). Nevertheless, it is generally recognized that the infections discussed here have resulted from contaminated heroin, which might have become contaminated during processing, transport, or storage. In the United Kingdom, 90% of heroin used originates in Afghanistan, where the opium is produced and—increasingly since 2002—converted to heroin. Heroin from Afghanistan usually travels over land, passing through several countries before entering the European Union and reaching the United Kingdom (*17*,*18*). The conditions in which heroin is processed, transported, and stored are uncertain; because these activities are illegal, they all probably make the drug vulnerable to inadvertent contamination with bacterial spores, for example, from soil or dust. Contaminated heroin is thought to have been the source of *B. anthracis* infection in a drug injector in Norway in 2000 (*19*,*20*) and in the more recent outbreak among PWID in Europe (*12*). Another source of potential contamination is drug adulterants (cutting agents), which are widely used to dilute and increase the bulk of illicit drugs (*21*). Although most infections probably resulted from upstream (before it reaches the end user) contamination of heroin, spores on the soiled hands of users and dirty needles could be inoculated during the injection process (*22*). This mode of infection remains unproven, although signs of tetanus were observed by Arthur Nicolaier in 1884 after he injected garden soil containing *C. tetani* (at that point unnamed) into animals (*23*), and clostridial infections after injection through dirt-covered hides have been reported (*24*).

Although the presence of bacterial spores is a necessary prerequisite for infection, several other factors might influence the development and geographic patterns of infections. The clustering of cases of *C. novyi *infection; anthrax; and, to a lesser extent, tetanus into outbreaks suggests that the contamination might have affected specific batches of heroin. By contrast, the botulism cases were generally more sporadic (albeit with some clustering) (*25,26*), suggesting that *C. botulinum* and, to a lesser degree, *C. tetani* spores might be more commonly present in the drug supply or in the local environment but at varying levels of contamination. Different drug supply routes serving eastern and western England and Scotland (*12*) might account for some of the geographic patterns and are consistent with the excessive *C. novyi* infections among PWID in Greater Glasgow and Clyde (western Scotland) and the North West region of England and with the higher rates of botulism among PWID in the East of England and Grampian (eastern Scotland).

Practices such as skin or muscle popping (intentionally or accidentally injecting into skin or muscle) (*10*,*27*,*28*) or the use of large amounts of citric acid to dissolve heroin can damage soft tissue, leading to necrosis and providing a suitable environment for anaerobic bacteria, such as *Clostridium* spp., to thrive. Older age (a proxy for a longer injecting career) and female sex have been associated with infections and injuries at injecting sites (*29*,*30*), which are associated with difficulty accessing veins. These persons might resort to injecting into the skin or muscle. Geographic variation in these practices might explain some of the variations seen in this study. PWID across England and Scotland might be regularly exposed to botulism and tetanus spores, but the levels of infection might be higher in some areas where skin or muscle popping is more common. This finding is consistent with the high proportion of women and the older median age among PWID with clostridial or *B. anthracis* infections described here in comparison with the wider population of PWID in England and Scotland. The emergence of these infections as a major public health issue in the United Kingdom and Ireland (*6*) over the past decade might reflect the changing characteristics of the drug-using population, an aging cohort of users resulting from the marked increase in injection drug use during the 1980s and 1990s (*31*). With regard to tetanus, variation could also reflect differences in the levels of effective immunization among PWID.

This analysis captures only the anthrax cases reported before the end of December 2009; however, the anthrax outbreak continued into 2010 and resulted in a total of 52 confirmed cases, including 5 in England (*12*,*32*). The risk factors for anthrax might differ from those for the other infections/diseases because anthrax is the only disease considered here that is caused by an aerobic bacterium. Furthermore, we cannot exclude the possibility of inhalational anthrax in some of the case-patients who reported smoking heroin (*11*,*12*). We considered only confirmed cases of anthrax in this analysis; however, the inclusion of probable cases (although it would have increased the numbers and rates) most likely would not have changed our findings with regard to demographic characteristics, given that probable and confirmed cases were similar in terms of age (mean 34 vs. 35 years, respectively) and sex (29% vs. 30% female, respectively) (*12*).

Because infections might go unreported or be misdiagnosed, the data presented here potentially underestimate the actual numbers of infections among PWID in England and Scotland. For tetanus and botulism, little toxin is required to cause symptoms; therefore, in combination with a reported history of injection drug use, index of clinical suspicion should be high (*33*–*35*). However, tetanus cases are underreported because some clinicians are not familiar with this rare disease (*36*). Misdiagnosis of infection might also account for underreporting because the symptoms of other illnesses can resemble those of the infections of interest in this study (e.g., Guillain-Barré syndrome vs. botulism) (*33*,*34*). In addition, if an injection site infection is treated promptly with broad spectrum antimicrobial drugs before tissue samples are collected, microbiological confirmation might not be possible (*7*).

Another limitation of this study is associated with estimates of the size of the PWID population. The estimates from Scotland and England were produced by using indirect methods by the same team but were based on different data sources and definitions. Moreover, estimates produced by indirect methods are difficult to validate. For example, the national study used here estimated 17,909 PWID in London (*13*), but another study estimated >30,000 PWID in London for 2000–2001 (*37*).

Because the quality and safety of illicit heroin is not monitored or controlled, sporadic cases and outbreaks of illness associated with spore-forming bacteria among PWID might continue. Persons who use heroin should be encouraged to seek treatment for their dependency. Health care professionals should educate PWID who continue to inject about injecting hygiene, the risks from specific injecting practices that have been associated with these infections, the need to ensure that their tetanus vaccinations are up to date, and the need to seek care if they have symptoms of an injection-related infection. Public health professionals should continue to be vigilant to ensure prompt detection of outbreaks and so permit the rapid dissemination of advice.
